# Accelerating pairwise statistical significance estimation for local alignment by harvesting GPU's power

**DOI:** 10.1186/1471-2105-13-S5-S3

**Published:** 2012-04-12

**Authors:** Yuhong Zhang, Sanchit Misra, Ankit Agrawal, Md Mostofa Ali Patwary, Wei-keng Liao, Zhiguang Qin, Alok Choudhary

**Affiliations:** 1 School of Computer Science and Engineering, University of Electronic Science and Technology of China, Chengdu, China; 2 Department of Electrical Engineering and Computer Science, Northwestern University, Evanston, USA

## Abstract

**Background:**

Pairwise statistical significance has been recognized to be able to accurately identify related sequences, which is a very important cornerstone procedure in numerous bioinformatics applications. However, it is both computationally and data intensive, which poses a big challenge in terms of performance and scalability.

**Results:**

We present a GPU implementation to accelerate pairwise statistical significance estimation of local sequence alignment using standard substitution matrices. By carefully studying the algorithm's data access characteristics, we developed a tile-based scheme that can produce a contiguous data access in the GPU global memory and sustain a large number of threads to achieve a high GPU occupancy. We further extend the parallelization technique to estimate pairwise statistical significance using position-specific substitution matrices, which has earlier demonstrated significantly better sequence comparison accuracy than using standard substitution matrices. The implementation is also extended to take advantage of dual-GPUs. We observe end-to-end speedups of nearly 250 (370) × using single-GPU Tesla C2050 GPU (dual-Tesla C2050) over the CPU implementation using Intel^© ^Core™i7 CPU 920 processor.

**Conclusions:**

Harvesting the high performance of modern GPUs is a promising approach to accelerate pairwise statistical significance estimation for local sequence alignment.

## Background

### Introduction

The past decades have witnessed dramatically increasing trends in the quantity and variety of publicly available genomic and proteomic sequence data. Dealing with the massive data and making sense of them are big challenges in bioinformatics [1,2]. One of the most widely used procedures for extracting information from proteomic and genomic data is pairwise sequence alignment (PSA). Given two sequences, PSA finds the extent of similarity between them. Many bioinformatics applications have been developed based on pairwise sequence alignment, such as BLAST [3], PSI-BLAST [4-6], and FASTA [7]. PSA produces a score for an alignment as a measure of the similarity between two sequences. Generally, the higher the score, the more related the sequences. However, the alignment score depends on various factors such as alignment methods, scoring schemes, sequence lengths, and sequence compositions [8,9]. Judging the relationship between two sequences solely based on the scores can often lead to wrong conclusion. Therefore, it is more appropriate to measure the quality of PSA using the statistical significance of the score rather than the score itself [10,11]. Statistical significance of sequence alignment scores is very important to know whether an observed sequence similarity could imply a functional or evolutionary link, or is a chance event [8,12]. Accurate estimation of statistical significance of gapped sequence alignment has attracted a lot of research in recent years [13-26].

### Pairwise statistical significance

Consider a pair of sequences *s*_1 _and *s*_2 _of lengths *m *and *n*, respectively, the scoring scheme, *SC *(substitution matrix, gap opening penalty, gap extension penalty), and the number of permutations *N *of *s*_2_, pairwise statistical significance (PSS) of the two sequences is calculated by the following function [21], which is described below:

$$PSS\left(s_{1},s_{2},m,n,SC,N\right),$$

Through permuting *s*_2 _*N *times randomly, the function generates *N *scores by aligning *s*1 against each of the *N *permuted sequences and then fits these scores to an extreme value distribution (EVD) [8,27,28] using censored maximum likelihood [29]. The returned value is the pairwise statistical significance of *s*_1 _and *s*_2_. The EVD describes an approximate distribution of optimal scores of a gapless alignment [4]. Based on this distribution, the probability (i.e., *P-value*) of observing an sequence pair with a score *S *greater than *x*, can be given by:

(1) $$P\left(S>x\right)\approx 1-exp\left(-Kmne^{-\lambda x}\right)=1-e^{-E\left(x\right)}$$

where *λ *and *K *are calculational constants and *E*(*x*), also known as *E-value*, is the expected number of distinct local alignments with score values of at least *x*.

Note that the above distribution is for a gapless alignment. For the cases of gapped alignment, although no asymptotic score distribution has yet been established analytically, computational experiments strongly indicate these scores still roughly follow Gumbel law [8,13,30].

In addition to not needing a database to estimate the statistical significance of an alignment, pairwise statistical significance is shown to be more accurate than database statistical significance reported by popular database search programs like BLAST, PSI-BLAST, and SSEARCH [21]. However, it involves thousands of such permutations and alignments, which are enormously time consuming and can be impractical for estimating pairwise statistical significance of a large number of sequence pairs. Hence, use of high-performance computing (HPC) techniques (such as multi-cores CPU, many-core graphics processing units (GPUs), FPGAs, etc.) is highly conducive to accelerate the computation of PSSE. Moreover, large data sets demand more computing power. In many demanding bioinformatics applications, such as sequence alignment [31,32], and protein sequence database search [33,34], many-core GPU has demonstrated its extreme computing power. This strongly motivates the use of GPUs to accelerate the PSSE. Acceleration of PSSE using MPI [35] and FPGA [36] has been explored earlier, and in this work, we design a GPU implementation for the same. Compared to [35,36], we consider the estimation of multi-pair PSS along with single-pair PSS using GPUs.

### Contributions

We present a GPU implementation to accelerate pairwise statistical significance estimation of local sequence alignment using standard substitution matrices. Our parallel implementation makes use of CUDA (Compute Unified Device Architecture) parallel programming paradigm. Our design uses an efficient data reorganization method to produce coalesced global memory access, and a tiled-based memory scheme to increase the GPU occupancy, a key measure for GPU efficiency [37]. Through careful analysis of the data dependency and access patterns of PSSE, we reorganize the data sequences into aligned arrays that coalesce the global memory access pattern. Such data access contiguity keeps the GPU cores occupied with computation and allows the thread scheduler to overlap the global memory access with the computation. In addition to the ability to calculate the optimal tile size for data to be shipped to the GPU, our design can also issue a large enough number of threads to maximize the occupancy.

We further extend the parallelization technique to estimate pairwise statistical significance using position-specific substitution matrices, which has earlier demonstrated significantly better sequence comparison accuracy than using standard substitution matrices [11]. The implementation is also extended to take advantage of dual-GPUs to accelerate those computations. As a result, maximum performance could be obtained by harvesting the power of many-core GPUs.

The performance evaluation was carried out on NVIDIA Telsa C2050 GPU. For multi-pair PSSE implementation, we observe nearly 250 (370)× speedups using a single-GPU Tesla C2050 GPU (dual-Tesla C2050) over the CPU implementation using an Intel^© ^Core ™i7 CPU 920 processor. The proposed optimizations and efficient framework for PSSE, which have direct applicability to speedup homology detection, are also applicable to many other sequence comparison based applications, such as DNA sequence mapping, phylogenetic tree construction and database search especially in the era of next-generation sequencing.

## Methods

In this section we present GPU implementations both for single-pair PSSE and multi-pair PSSE. Along with the methodological details, we also discuss several performance optimization techniques used in this paper, such as, GPU memory access optimization, occupancy maximization, and substitution matrix customization. These techniques have significantly speeded up PSSE.

Careful analysis of the data pipelines of PSSE shows that the computation of PSSE can be decomposed into three computation kernels: Permutation, Alignment and Fitting. Permutation and Alignment comprise the overwhelming majority (a more than 99.8%) of the overall execution time [35]. Therefore, efforts should be spent to optimize these two kernels to achieve high performance. Also, we observe that permutation presents high degrees of data independency that are naturally suitable for single-instruction, multiple-thread (SIMT) architectures [38] and therefore, can be mapped very well to task parallelism models of GPU. Moreover, even though the alignment task suffers from data dependency, we show that with clever optimizations, it can be heavily accelerated using GPUs.

### Design

#### GPU memory access optimization

It is especially important to optimize global memory access as its bandwidth is low and its latency is hundreds of clock cycles [38]. Moreover, global memory coalescing is the most critical optimization for GPU programming [39]. Since the kernels of PSSE usually work over large numbers of sequences that reside in the global memory, the performance is highly dependent on hiding memory latency. When a GPU kernel is accessing global memory, all threads in groups of 32 (i.e. *warp*) access a bank of memory at one time. A batch of memory accesses is considered coalesced when the data requested by a warp of threads are located in contiguous memory addresses. For example, if the data requested by threads within a warp are located in 32 consecutive memory addresses (such that the *i^th ^*address is accessed by the *i^th ^*thread), the memory can be read in a single access. Hence, this memory access operation runs 32 times faster. If the memory access is not coalesced, it is divided into multiple reads and hence serialized [37].

**Figure 1:**
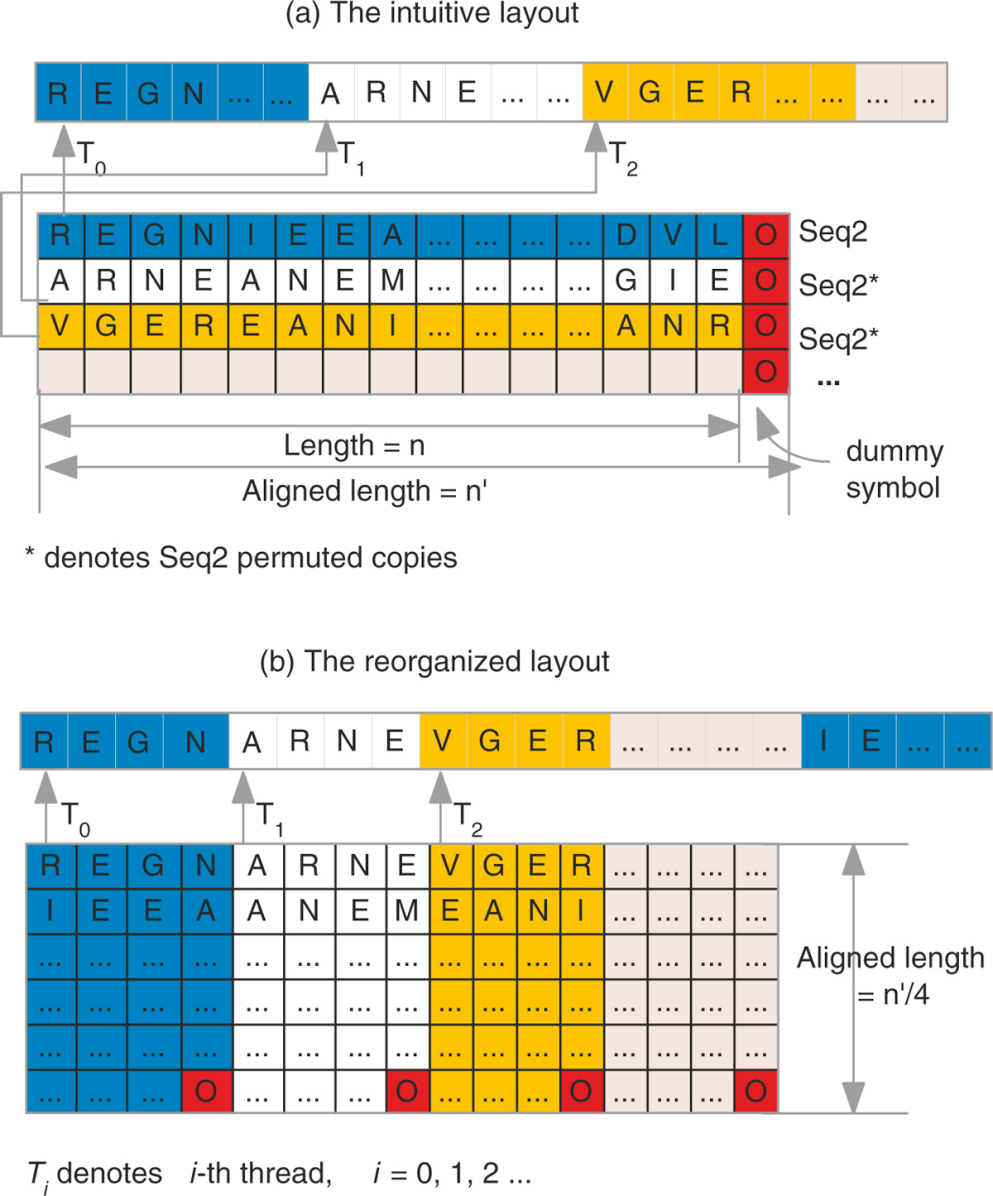
**Two GPU memory layout strategies**. (a) The intuitive layout that one sequence is appended after another. (b) The reorganized layout such that the uchar4 at the same indices in different sequences stay at neighboring positions.

After permutation, if the sequence *s*_2 _and its *N *permuted copies were stored contiguously one after another in the global memory, the intuitive memory layout would be as shown in Figure 1 (a) Note that, we need one byte (*uchar*) to store each amino acid residue. Moreover, GPU can read four-byte (packed as a CUDA built-in vector data type *uchar4*) of data from the global memory to registers in one instruction. To achieve high parallelism of global memory access, *uchar4 *is used to store the permuted sequences. Dummy amino acid symbols are padded in the end to make the length of sequences a multiple of 4.

Considering inter-task parallelism, where each thread works on the alignment of one of the permuted copies of *s*_2 _to *s*_1_, in this layout the gap between the memory accesses by the neighboring threads is at least the length of the sequence. For example, in the intuitive layout, if thread *T*_0 _accesses the first residue (i.e., 'R'), and thread *T*_1 _accesses the first residue (i.e., 'E'), the gap between the access data is *n*. This results in non-coalesced memory reads (i.e., serialized reads), which significantly deteriorates the performance.

We therefore reorganize the layout of sequence data in memory to obtain coalesced reads. Now, to achieve coalesced access, we reorganize layout of sequences in memory as aligned structure of arrays, as shown in Figure 1 (b) In the optimized layout, the characters (in granularity of 4 bytes) that lie at the same index in different permuted sequences stay at neighboring positions. Then if the first *uchar4 *of the first permuted sequence (i.e. 'REGN') is requested by thread *T*_0_, the first *uchar4 *of the second permuted sequence (i.e. 'ARNE') is requested by *T*_1_, and so on. This results in reading a consecutive memory (each thread reads 4 bytes) by a warp of threads in a single access. Thus the global memory access is coalesced, and therefore high performance is achieved.

As the sequences remain unchanged during the alignment, they can be thought of as read-only data, which can be bound to texture memory. For read patterns, texture memory fetches is a better alternative to global memory reads because of texture memory cache, which can further improve the performance.

#### Occupancy maximization

Hiding global memory latency is very important to achieve high performance on the GPU. This can be done by creating enough threads to keep the CUDA cores always occupied while many other threads are waiting for global memory accesses [39]. GPU *occupancy*, as defined below, is a metric to determine how effectively the hardware is kept busy:

(2) $$Occupancy=\left(B\times T_{num}\right)/T_{max}$$

where *T_max _*is maximum number of resident threads that can be launched on a streaming multiprocessor (SM) (which is a constant for a specific GPU), *T_num _*is the number of active threads per block and *B *is the number of active blocks per Streaming Multiprocessor (SM).

*B *also depends upon the GPU physical limitations (e.g. the amount of registers, shared memory and threads supported in each model). It can be given in the following way:

(3) $$B=\text{min}\left(B_{user},B_{reg},B_{shr},B_{hw}\right)$$

where *B_hw _*is the hardware limit (only 8 blocks are allowed per SM), and *B_reg_*, *B_shr_*, are the potential blocks determined by the available registers, shared memory, respectively and *B_user _*is blocks set by the user. Note that *B *≤ *B_hw_*, therefore we obtain:

(4) $$Occupancy\leq \left(B_{hw}\times T_{num}\right)/T_{max}$$

Based on the above analysis, higher occupancy can be pursued according to the following rules:

• To avoid wasting computation on under-populated warps, the number of threads per block should be chosen as a multiple of the warp size (currently, 32).

• Total number of active threads per SM should be close to maximum number of resident threads per multiprocessor (*T_max_*). In short, we should use all the available threads if possible.

• To achieve 100% occupancy, (*B_hw _*× *T_num_*)/*T_max _*≥ 1. Hence, setting *T_num _*such that *T_num_*≥ *T_max_*/*B_hw _*is preferable.

#### Substitution matrix customization

**Figure 2:**
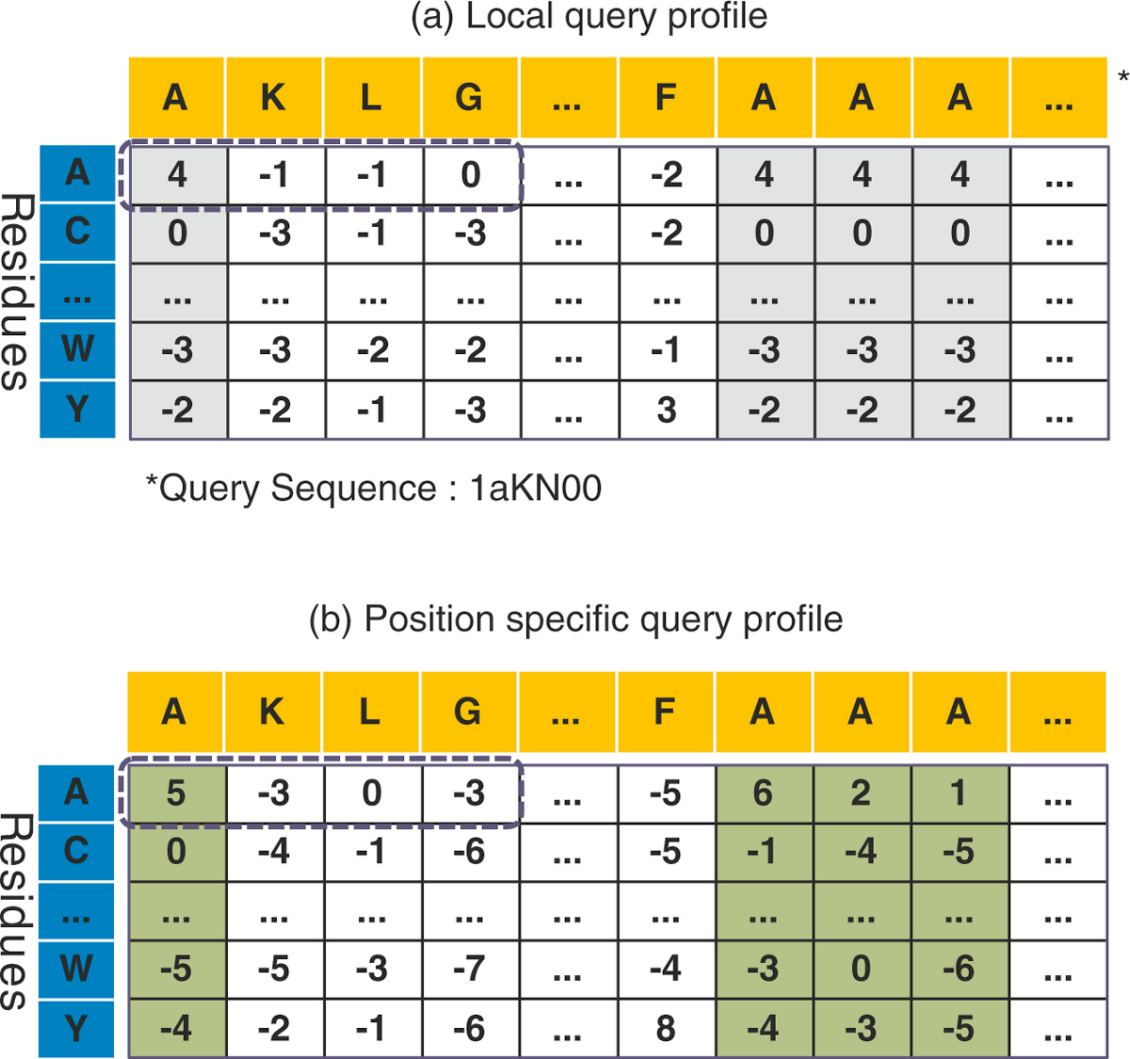
**The customized substitution matrix**. (a) Local query profile: the same symbol (e.g., 'A') at different position in query has same scores. (b) Position specific query profile: the same symbol (e.g., 'A') at different position in query has different scores.

The Smith-Waterman (SW) algorithm [40] looks up the substitution score matrix very frequently while computing the alignment scores. Lowering the number of lookups obviously reduces the overall execution time. As suggested by [41], performance improvement could further be obtained by customizing the matrix. Usually, the substitution score matrix is indexed by query-sequence symbol and the subject-sequence symbol as in BLOSUM62. Another way is to index the substitution score matrix by query-sequence position and the subject-sequence symbol. We refer to this customized substitution matrix as *local query profile*. For example, let us consider a query sequence *Q *of length *L *over a set of residue alphabet *ω*. For each residue, we store a substitution score for every query sequence position. For example, in Figure 2 (a) the substitution scores for matching residue 'A' with each symbol of the query sequence *Q *are stored in the first row, and the substitution scores for matching residue 'C' are stored in the next row, and so on. Here the substitution score of the residue of subject sequence against the same symbol of query sequence at different position is always same.

By contrast, position-specific score matrix (PSSM) [4] offers a variation of this approach. We call this a *position specific query profile *in which scores are further refined. In this case the same residue (e.g. 'A') appearing at different position of query sequence has different scores, as shown in Figure 2 (b). For a given query sequence, PSSM can be pre-constructed by PSI-BLAST [3,4].

Using the customized approach, in both cases, the dimension |*ω*| × |*ω*| of the substitution matrix is replaced by a query-specific matrix of dimension |*ω*|×|*L*|. This increases the memory requirement compared to the original layout, but reduces the lookups of the substitution matrix significantly as explained below.

In traditional matrix layout, if the substitution scores of a subject sequence residue (i.e., 'A') against query sequence residues (i.e., 'A','K','L','G') are required subsequently, GPU has to look up the matrix four times one by one, because the score *S*[*A*][*A*], *S*[*A*][*K*], *S*[*A*][*L*], *S*[*A*][*G*] are usually stored far from each other. In contrast, if we use CUDA built-in vector data type *int4 *(which packs 4 integers together) to store the customized substitution matrix in the texture memory, the same four scores are stored at neighboring memory locations (as shown in Figure 2 (b). By reading an *int4*, the GPU can get these four scores *S*[*A*][*i*] in one instruction, where *i *is the position of residues in query sequence. This reduces the number of lookups by a factor of 4, therefore, a higher performance can be obtained.

In essence, both coalesced memory layout and customized substitution matrix optimization heighten performance of memory access by improving data locality among threads in GPU programming.

### Single-pair PSSE implementation

As mentioned previously, to simulate the required random sequences, a lot of random numbers are needed, since each *s*_2 _copy has to be permuted thousands of times. Although the most widely used pseudorandom number generators such as linear congruential generators (LCGs) can meet our requirements, the current version of CUDA does not support calls to the host random function. Hence, we develop an efficient random number generator similar to *lrand48*() on GPUs.

The single-pair PSSE processes only one pair of query and subject sequences. The idea of computing single-pair PSSE is as follows. Given the query sequence *s*_1 _and the subject sequence *s*_2_, to compute PSSE, thousands (say *N*) of randomly permutations of *s*_2 _are needed. To obtain these *N *random sequences of *s*_2_, first, a set of *N *random numbers is generated in CPU as described in previous section. These numbers are then transferred to GPU and considered as seeds by the threads of GPU. Each thread then generates new random numbers using its own seed and swap the symbols of *s*_2 _accordingly to obtain a permuted sequence. Thus the *N *random permutations of *s*_2 _are obtained in parallel. The algorithm then uses SW algorithm to compute alignment score of *s*_1 _and the *N *permuted copies of *s*_2 _in parallel on GPU. The scores are then transferred to CPU for fitting. The details of computing single-pair PSSE is outlined by Algorithm 1. Note that Step 4 in Algorithm 1 uses the optimized memory layout, explained previously in detail, for *s*_2 _and its *N *permuted copies.

Smith-Waterman is a dynamic programming algorithm to identify the optimal local alignment between a pair of sequences. In general, there are two different methods for parallelizing the alignment task [33]. The first method is regarded as *inter-task parallelism*. In this case, each thread performs alignment of one pair of sequences. Hence, in a thread block, multiple alignment tasks are performed in parallel [42]. The second one is *intra-task parallelism*. Here, alignment of each pair of sequences is assigned to a block of threads, splitting the whole task into a number of sub-tasks. Each thread in the thread block then performs its own sub-tasks, cooperating to exploit the parallel characteristics of cells in the anti-diagonals of the local alignment matrix [43]. We use a similar alignment kernel as proposed in [34] with some modifications for further improvement. It is worthwhile to mention that Step 7 describes fitting, which is implemented on the CPU. This is because it involves recursion that is not supported very well on GPU.

### Multi-pair PSSE implementation

The multi-pair PSSE processes multiple queries and subject sequences. We can obtain some hints about improving the performance by analyzing the single-pair PSSE implementation (which we do in a later section). Owing to a dramatic increase of data set for multiple-pairs implementation, there are some significant differences relative to performance of GPU hardware between the two implementations. Through analyzing our experimental results of the single-pair PSSE, we observe that inter-task parallelism performs better than intra-task parallelism (results shown later). Hence, we consider inter-task parallelism in computing multi-pair PSSE. Based on the guidelines for optimizing memory and occupancy described earlier, we compare three implementation strategies.

**Algorithm 1: **Pseudo-code of single-pair PSSE

**Input**: (*s*_1_, *s*_2_) - Sequence-pair; *M *- Substitution matrix; *G *- Gap opening penalty; *GE *- Gap extension penalty; *N *- Number of permutes;

**Output**: *pss*- Pairwise statistical significance

1. Initialization

(a) Generate a number *N *of random numbers in CPU;

(b) Copy LCG seeds to GPU global memory;

(c) Copy *s*_1 _and *s*_2 _to GPU global memory;

2. Generate random numbers (RNs) using LCG in GPU

3. Permute sequence *s*_2 _*N *times using the RNs

4. Reorganize sequences *s*_2 _and its *N *times permuted copies as shown in Figure 2 (b) if using inter-task parallel Smith-Waterman();

5. Align *s*_2 _and its *N *permuted copies against *s*_1 _using Smith-Waterman() (*inter-task *or *intra-task*);

6. Transfer *N *alignment scores from GPU into CPU;

7. Fitting in CPU

(a) (*K*, *λ*) ← *EV DCensordMLFit*(*Scores*);

(b) *pss *← 1 - *exp*(-*Kmne *-*^λx^*);

**return ***pss*

#### Intuitive strategy

Given *Q *query sequences and *S *subject sequences, the intuitive strategy is to simply perform the same 'single-pair' procedure *Q *× *S *times. In other words, in each iteration, we send a single pair of query and subject sequences to the GPU. The GPU processes that pair and returns the result to the CPU. The same procedure is repeated for all query and subject sequence pairs. However, this strategy suffers from low occupancy. We analyze the cause along with its performance results in the next section.

#### Data reuse strategy

In the first strategy, the subject sequences from the database are permuted for every query-subject sequence pair. Hence, the same subject sequence is permuted every time it is sent to the GPU. A better strategy is to create permutations of each subject sequence only once and reuse them to align with all the queries. "One permutation, all queries" is the idea of the second strategy. Because of the reuse of permuted sequences, higher performance is expected than the first strategy. However, the occupancy of GPU, to be shown in the next section, is still not elevated.

#### Adaptively tile-based strategy

The low occupancy of the above two strategies is due to the underutilized computing power of GPU. In addition, these two strategies do not work well when the size of subject sequence database becomes too big to be fitted into GPU global memory. For instance, if the size of the original subject sequence database is 5 MB, it becomes 5000 MB when each of the sequences is permuted, say, 1000 times. This prohibits transfer of all the subject sequences to GPU at the same time. We therefore need an optimal number of subject sequences to be shipped to GPU keeping in mind that the subject sequences and their permuted copies fit in global memory. Moreover, the number of new generated sequences should be enough to keep all CUDA cores busy, i.e., keep a high occupancy of GPU, which is very important to harness the GPU power.

Herein we develop a memory tiling technique that is self-tuning based on the hardware configuration and can achieve a close-to-optimal performance. The idea behind the technique is as follows. In out-of-core fashion, the data in the main memory is divided into smaller chunks called *tiles *and transferred to the GPU global memory. In our case, the tiles are the number of subject sequences to be transferred to the GPU at a time. The tile size *T *can be calculated using the following equation:

(5) $$T=\left\lfloor \frac{SM_{num}\times T_{max}}{N}\right\rfloor$$

where *SM_num _*is the total number of SMs in GPU, *T_max _*is the maximum number of resident threads per SM, and *N *is the number of permutations.

**Figure 3:**
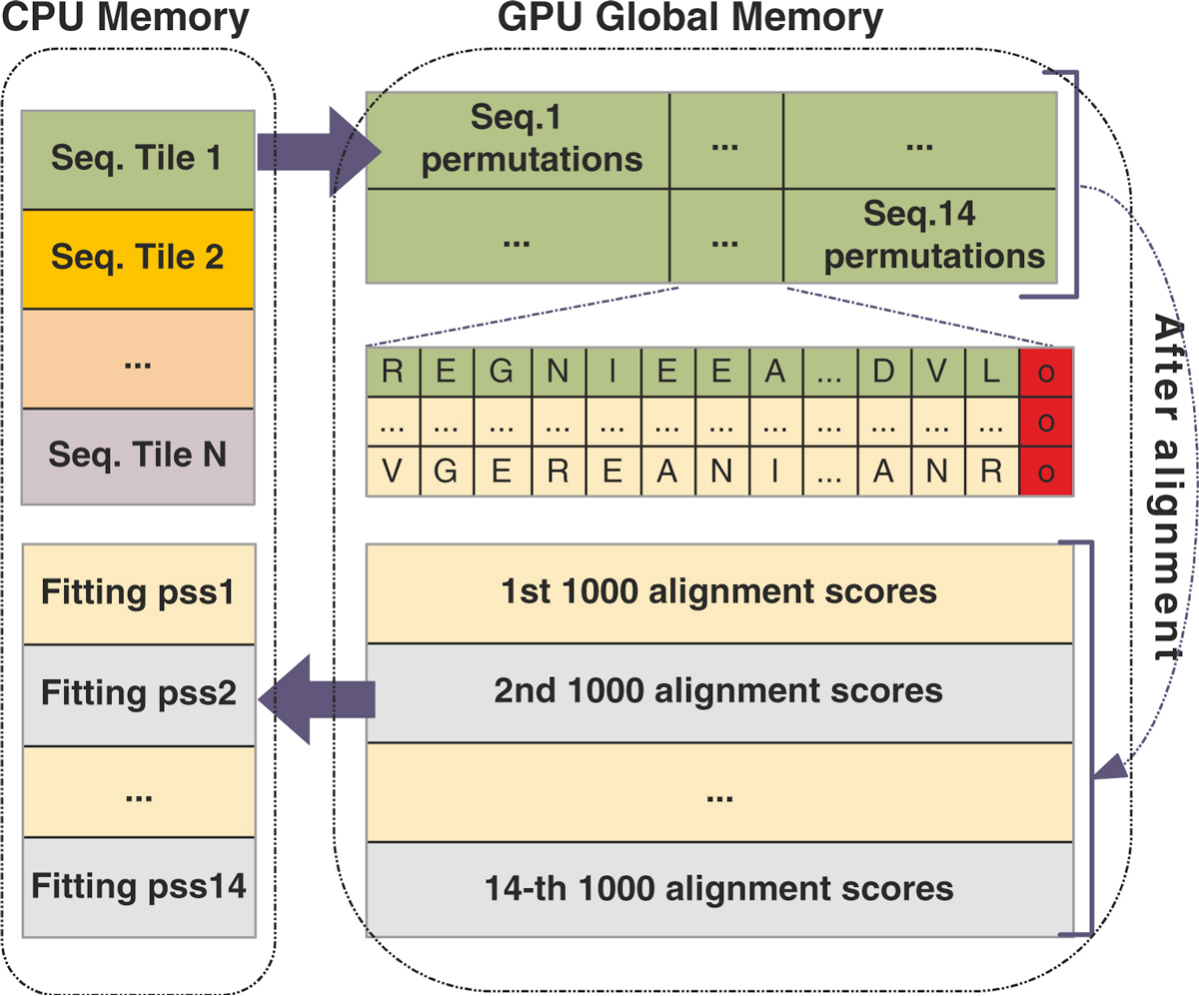
**Adaptive tile-based strategy**. The optimal tile size *T *can be calculated according to the hardware configuration of GPU. After calculated, the *T *subject sequences together are transferred to GPU global memory. Permutations and alignments are done in parallel in GPU. Then *T *× 1000 alignment scores are moved back to CPU for *T *fittings.

In Tesla C2050 used in our experiments, there are 14 SMs and the maximum number of resident threads per SM is 1024. Let *N *= 1000, then, *T *= ⌊14 × 1024/1000⌋ = 14, which means that there are 14 distinct subject sequences to be transferred to the GPU's global memory at a time. Based on the second strategy (i.e., data reuse), 14 subject sequences and their permuted copies are aligned against one query sequence at a time, until all the query sequences are processed. As a result, 14 × 1000 alignment scores in total are obtained in each round, which are subsequently transferred to CPU for fitting. The CPU takes the 1000 alignment scores for each subject-query sequence pair and uses them to compute the corresponding *pss*. The tile-based strategy has been described in Figure 3.

## Results and discussion

All our experiments have been are carried out using Intel^© ^Core™i7 CPU 920 processors running @2.67 GHz. The system has 4 cores, 4 GB of memory, dual Tesla C2050 GPU (each with 448 CUDA cores) and is running a 64-bit Linux-based operating system. Our program has been compiled using gcc 4.4.1 and CUDA 4.0. The sequence data used in this work comprises of a non-redundant subset of the CATH 2.3 database [44]. This dataset consists of 2771 domain sequences as our subject library and includes 86 CATH queries as our query set. We derive PSSMs for the 86 test queries against the non-redundant protein database using PSI-BLAST (provided along with the BLAST+ package [3]) over a maximum of five iterations and with other default parameters. BLOSUM62 and PSSMs have been used as the scoring matrices with affine gap penalty of 10 + 2*k *for a gap of length *k*. We permute the 2771 subject sequences *N *= 1000 time as in several previous studies [21,35,36,45].

### Single-pair PSSE result analysis

**Figure 4:**
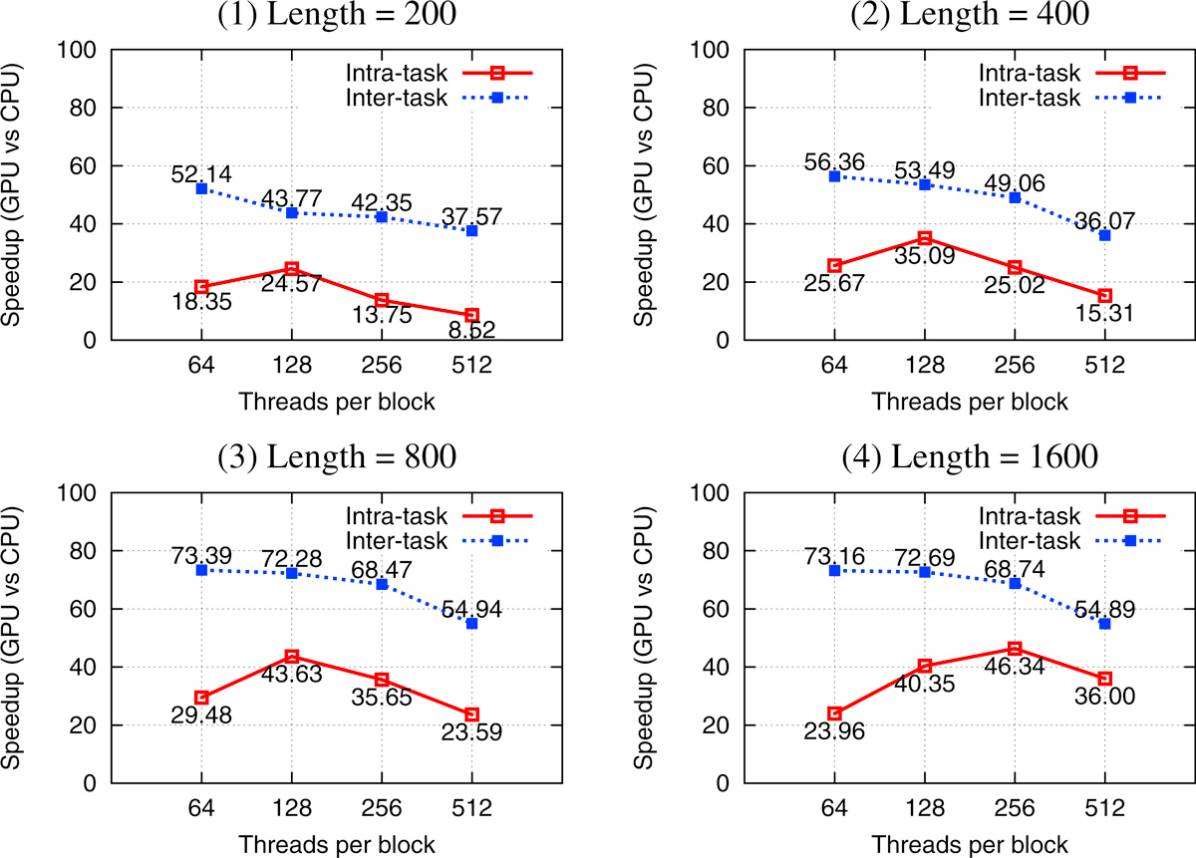
**Intra-task and inter-task parallelism for single-pair PSSE**. We choose four pairs of query and subject sequences of length 200, 400, 800, and 1600 from CATH database.

In single-pair PSSE implementation, we use both the intra-task and inter-task parallelism methods. We choose four pairs of query and subject sequences of length 200, 400, 800, and 1600 from CATH 2.3 database. We compute PSSE for all these pairs using 64, 128, 256, and 512 threads per block. The experimental results have been plotted in Figure 4. All speedups are computed over the corresponding the CPU implementation. We observe that the inter-task parallel implementation performs significantly better than the intra-task parallel implementation. Employing further optimizations and newer version of CUDA, both methods show a higher speedup compared to our previous results [31].

For intra-task parallel implementation, the best speedups for sequences of length 200, 400, and 800 are 24.57×, 35.09×, and 43.63×, respectively. All these are obtained using 128 threads per block. But the best speedup for sequences of length 1600 is 46.34× and used 256 threads per block. These results tell us that it is hard to find a general rule to parameterize the settings to achieve peak performance. A possible reason is that many factors, such as the number of threads per block, the available registers, and shared memory, may contradict with each other.

The intra-task parallel implementation creates enough thread blocks (one block for each alignment task) to keep the occupancy high. However, the SW alignment matrix must be serially computed from the first to the last anti-diagonal. Only the cells of the alignment matrix belonging to the same anti-diagonal can be computed in parallel. In this case, most threads in a block have no work to do. As a result, the performance of the intra-task parallel implementation is worse than that of the inter-task parallel implementation even though it has higher occupancy.

In contrast, inter-task parallelism would have a total of 1000 threads (one for each alignment task). If each block contains 64 threads, then the total number of blocks is *B *= ⌈1000/64⌉ = 16. The assignment of 16 blocks to 14 available SMs will result in 12 SMs with one active block and two SMs with two blocks. Therefore, the occupancy for the two SMs with two blocks is (2 × 64)/1024 = 12.5%. For the 12 SMs with only one block its occupancy is (1 × 64)/1024 = 6.25%. Because of the low occupancy, there are not sufficient threads to keep CUDA cores busy when the global memory is accessed. As a result, the latency-hiding capabilities of this method are limited. As the number of threads per block *T_num _*increases, the total active blocks (*B*) decreases and some SMs even have no blocks assigned. For example, if *T_num _*= 512, then *B *= ⌈1000/512⌉ = 2. Hence only two SMs are working with one block each.

However, these SMs have a higher occupancy (1 × 512)/1024 = 50%, which compensates for the decrease in the number of working SMs. Consequently, even though there is a reduction in speedups, it is not directly proportional to the reduction in the number of active SMs. When the number of threads per block is 64, for the sequence length of 200, 400, 800, and 1600, the best speedups are 52.14×, 56.36×, 73.39×, and 73.16×, respectively.

In brief, getting performance out of a GPU is about keeping the CUDA cores busy. Both inter-task parallelism and intra-task parallelism, under the situations of small data set being processed, seem to fail in this regard, but not necessarily for the same reason.

### Multi-pair PSSE result analysis

**Figure 5:**
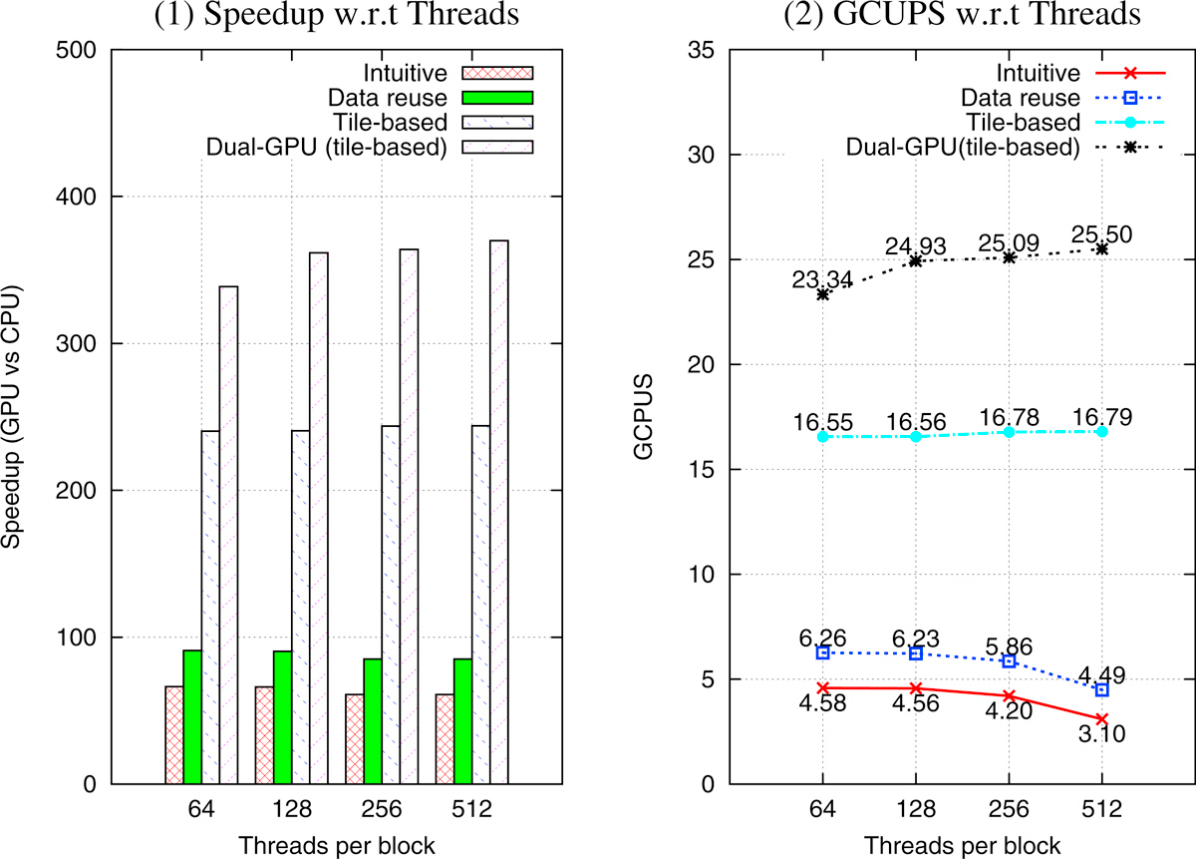
**Performance of three strategies for multi-pair PSSE**. All experiments are run using 2771 subject sequences and 86 query sequences from the CATH 2.3 database.

In the intuitive and data reuse strategies, most of the SMs suffer from the same low occupancy as the single-pair PSSE implementation using the inter-task parallelism method. As expected, we observe poor performance for both strategies, as shown in Figure 5. The data reuse strategy produces higher performance than the intuitive strategy, because the number of permutations is reduced by a factor of *Q*, the number of query sequences. To alleviate the low occupancy problem, our proposed tile-based strategy uses a carefully tuned tile size that effectively increase the occupancy. Recall that, as a result of tiling, we send 14 subject sequences and one query sequence to the GPU in each round. After the permutation step, we have 14 × 1000 alignments to be performed, assuming each subject sequence is permuted 1000 times. Consequently, each SM has 1000 alignments to perform. Also, note that, the maximum number of blocks that can be launched simultaneously on an SM is 8. Therefore, when the number of threads per block is 64, the number of blocks per SM is *min*(⌈1024/64⌉, 8) = 8, resulting in an occupancy of (8 × 64)/1024 = 50%, based on Equation (2) and (3). This is a significant improvement over the intuitive and data-reuse strategies. The maximum theoretical occupancy of an SM for the three strategies is given in Table 1.

**Table 1:** The maximum occupancy for three strategies

Threads/blocks	64	128	256	512
Intuitive occupancy	12.5%	6.25%	6.25%	6.25%
Data-reuse occupancy	12.5%	6.25%	6.25%	6.25%
Tile-based occupancy	50%	100%	100%	100%

Due to a high occupancy, the tile-based strategy using single (dual) GPU(s) achieves speedups of 240.31 (338.63)×, 243.51 (361.63)×, 240.71 (363.99)×, and 243.84 (369.95)× using 64, 128, 256, and 512 threads per block, respectively, as shown in Figure 5.

The billion cell updates per second (GCUPS) value is another commonly used performance measure in bioinformatics [33]. The tile-based strategy using single (dual) GPU(s) achieves performance results in the range of 16.55 (23.34) to 16.79 (25.50) GCUPS, as shown in Figure 5. Although a direct comparison across different GPU implementations and hardware in not fair, just for the sake of completeness, we cited below the performance in GCUPS reported by some existing implementations of the Smith-Waterman alignment task. It is worthwhile to mention here that [46] has a peak performance of 4.65 to 8.99 GCUPS for various query lengths on an NVIDIA 9800 and [42] has a peak performance of 3.5 GCUPS on a workstation running two GeForce 8800 GTX. Our implementation show higher GCUPS.

In summary, low occupancy is known to interfere with the ability to hide latency on memory-bound kernels, causing performance degradation. However, increasing occupancy does not necessarily increase performance. In general, once a 50% occupancy is achieved, further optimization to gain additional occupancy has little effect on performance [37]. Our experiments verify this claim.

Since the GPU implementation presented in this paper uses the same algorithm for PSSE (specifically the Smith-Waterman algorithm for getting alignment scores, the same fitting routine to get statistical parameters *K *and *λ*, and the same algorithm parameters) as in [11], the retrieval accuracy of the proposed implementation is expected to be identical to [11]. According to [11], pairwise statistical significance with standard substitution matrices performs at least comparable or significantly better than database statistical significance (using BLAST, PSI-BLAST, and SSEARCH). Moreover, pairwise statistical significance with PSSMs performs significantly better than using standard substitution matrices, and also better than PSI-BLAST using pre-constructed position-specific substitution matrices. More details can be found in [11]. The implementation of the proposed method and related programs in CUDA is available for free academic use at http://cucis.ece.northwestern.edu/projects/PSSE/.

## Conclusions

In this paper, we present a high performance accelerator to estimate the pairwise statistical significance of local sequence alignment, which supports standard substitution matrix like BLOSUM62 as well as PSSMs.

Our accelerator harvests the computation power of many-core GPUs by using CUDA, which results in high end-to-end speedups for PSSE. We also demonstrate a comparative performance analysis of single-pair and multi-pair implementations. The proposed optimizations and efficient framework are applicable to a wide variety of next-generation sequencing comparison based applications, such as, DNA sequence mapping and database search. As the size of biological sequence databases are increasing rapidly, even more powerful high performance computing accelerator platforms are expected to be more and more common and imperative for sequence analysis, for which our work can serve as a meaningful stepping stone.

## Abbreviations

BLAST: Basic Local Alignment Search Tool; BLOSUM: BLOcks of Amino Acid SUbstitution Matrix; CUDA: Compute Unified Device Architecture; EVD: Extreme Value Distribution; GCUPS: Billion Cell Updates Per Second; GPU: Graphics Processing Unit; PSSM: Position Specific Scoring Matrix; PSSE: Pairwise Statistical Significance Estimation; PSI BLAST: Position Specific Iterative BLAST; PSA: Pairwise Sequence Alignment; PSS: Pairwise Statistical Significance; SIMT: Single: Instruction, Multiple: Thread; SSM: Standard Substitution Matrix SM: Streaming Multiprocessor; SW: Smith-Waterman.

## Competing interests

The authors declare that they have no competing interests.

## Authors' contributions

YZ conceptualized the study, carried out the design and implementation of the algorithm, analyzed the results and drafted the manuscript; SM, AA, MMAP, WL, ZQ, and AC participated analysis of the results and contributed to revise the manuscript. All authors read and approved the final manuscript.
